# Phenotype–Genotype Correlations in Three Different Cases of Adult-Onset Genetic Focal Segmental Glomerulosclerosis

**DOI:** 10.3390/ijms242417489

**Published:** 2023-12-14

**Authors:** Tibor Kalmár, Sándor Turkevi-Nagy, László Bitó, László Kaiser, Zoltán Maróti, Dániel Jakab, Annamária Letoha, Péter Légrády, Béla Iványi

**Affiliations:** 1 Department of Pediatrics, Albert Szent-Györgyi Health Centre, University of Szeged, Temesvari krt 35-37, 6726 Szeged, Hungarymaroti.zoltan@med.u-szeged.hu (Z.M.);; 2 Department of Internal Medicine, Albert Szent-Györgyi Health Centre, University of Szeged, 6726 Szeged, Hungary; 3 Department of Internal Medicine, Centre of Clinical Infectology and Acute Internal Medicine, Albert Szent-Györgyi Health Centre, University of Szeged, 6726 Szeged, Hungary

**Keywords:** *ACTN4* gene, *CLCN5* gene, *COL4A5* gene, genetic FSGS, *PAX2* gene, proteinuria, renal biopsy

## Abstract

This study highlights the importance of a combined diagnostic approach in the diagnosis of rare diseases, such as adult-onset genetic FSGS. We present three adult patient cases evaluated with kidney biopsy for proteinuria, chronic kidney disease, and hypertension, which were suggestive of adult-onset genetic FSGS. Renal biopsy samples and formalin-fixed, paraffin-embedded fetal kidneys were evaluated using standard light microscopical stainings, direct immunofluorescence on cryostat sections, and electron microscopy. Clinical exome sequencing was performed for each case, and 45 FSGS-related genes were analyzed. Identifying mutations in the *PAX2*, *ACTN4*, and *COL4A5* genes have prompted a re-evaluation of the previous histopathological examinations. The *PAX2* mutation led to a thinner nephrogenic zone and decreased number of glomeruli, resulting in oligohydramnios during fetal development and oligomeganephronia and adaptive focal-segmental glomerulosclerosis in adulthood. The *ACTN4* mutation caused distinct electron-dense aggregates in podocyte cell bodies, while the *COL4A5* mutation led to segmental sclerosis of glomeruli with marked interstitial fibrosis and tubular atrophy. The identification of specific mutations and their histopathological consequences can lead to a better understanding of the disease and its progression, as well as potential treatment options.

## 1. Introduction

Focal segmental glomerulosclerosis (FSGS) is a light microscopical pattern of injury characterized by segmental obliteration of the glomerular capillary tufts caused by extracellular matrix accumulation (sclerosis) and/or deposition of hyaline material, often with the adhesion of tufts to Bowman’s capsule. The lesion affects less than 50% of glomeruli (focal). The clinical feature of FSGS is proteinuria with or without the nephrotic syndrome. Four etiological subsets of FSGS are currently classified: primary, secondary, genetic, and unclassifiable [1,2,3]. Primary FSGS is presumably caused by a still unidentified circulating podocyte-toxic permeability factor and typically manifests itself in the nephrotic syndrome, which may respond to corticosteroids. Secondary FSGS is commonly caused by adaptive structural–functional responses leading to glomerular hyperfiltration or viral infections, or certain drugs. Primary FSGS exhibits diffuse foot process effacement (FPE) with electron microscopy (EM), while adaptive FSGS displays both preserved and effaced foot processes [3].

Genetic FSGS may present as a sporadic or familial disorder, and it may be renal limited or syndromic. Over 50 FSGS-associated genes have been identified so far, most of which play a role in regulating the architecture and function of podocytes or the composition of the glomerular basement membrane (GBM) [3,4,5]. A typical manifestation is the steroid-resistant nephrotic syndrome in infants and young children, and its inheritance is autosomal recessive. Adults with an FSGS lesion may also have genetic FSGS, and the inheritance is usually autosomal dominant. The clinical appearance varies from patient to patient, and a fully blown nephrotic syndrome is unusual.

Here, we present three adult patients evaluated through kidney biopsy for proteinuria, chronic kidney disease, and hypertension. The clinical and biopsy features were suggestive of adult-onset genetic FSGS and prompted genetic testing with a targeted gene panel retrieved from clinical exome sequencing. The identified mutations in the *PAX2*, *ACTN4*, or *COL4A5* gene resolved the clinicopathologically puzzling cases. A detailed phenotype–genotype analysis of cases with adult-onset genetic FSGS is seldom published nowadays, so the unique features of presentations may be of interest to molecular biologists, clinical nephrologists, and nephropathologists.

## 2. Results

### 2.1. Case Presentations

Table S1 shows the synopsis of clinical data, and Table S2 summarizes the kidney biopsy findings.

### 2.2. Patient 1

He was evaluated in 2022 because of slowly worsening proteinuria, stage IV chronic kidney disease, slightly smaller kidneys (longitudinal diameter of the right kidney: 96 mm; left kidney: 100 mm), and hypertension. Two years earlier, the eGFR value was still 55 mL/min/1.73 m^2^. He was born in term, with a birth weight of 3000 g. He was severely obese from childhood. Although symptoms of renal disease and elevated blood pressure values were known for years, he agreed to the biopsy evaluation only a couple of weeks before the biopsy procedure and started to take the antihypertensive drugs prescribed. The family history of kidney disease was positive (Figure S1A).

#### 2.2.1. Renal Biopsy Findings and Genetic Analysis

**Figure 1:**
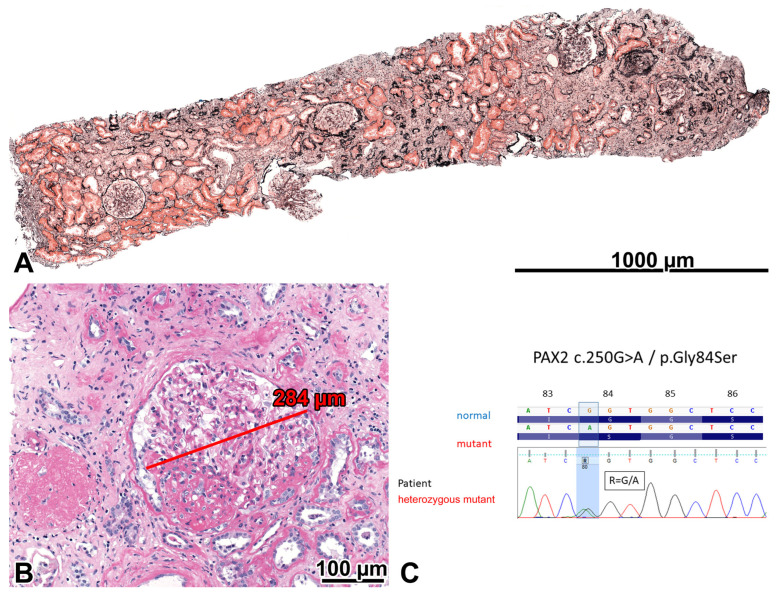
Oligomeganephronia and adaptive focal-segmental glomerulosclerosis (FSGS) in the severely obese patient with a *PAX2* mutation. (**A**) Five patent glomeruli and one globally sclerosed glomerulus can be seen in the 3 mm long cortical portion of the biopsy sample. The sparsity of glomeruli indicated that the number of glomeruli was lower than normal in the patient. Foci of interstitial fibrosis and tubular atrophy can be seen. Jones, ×10. (**B**) Two glomeruli are shown. One is globally sclerosed, and the other is markedly enlarged and exhibits a perihilar FSGS lesion. PAS, ×20. (**C**) A heterozygous *PAX2* mutation (exon 3; NM_003990.3:c.250G>A and NP_003981.2:p.Gly84Ser) was demonstrated, characterized by a single nucleotide change (c.250G>A). The Sanger sequence traces show the nucleotide change position (highlighted in blue).

The glomeruli were sparsely distributed and markedly enlarged, and they exhibited an FSGS lesion characterized by an occasional perihilar location, subendothelial hyaline deposits, and insudation of IgM and C3 on IF. Severe interstitial fibrosis and tubular atrophy, interstitial foamy cells, and eccentric subendothelial hyalinosis in afferent arterioles accompanied the glomerular lesions. The quantitative values of glomerular size and glomerular paucity supported our observational findings. One patent glomerulus was examined electron microscopically. Diffuse FPE was not seen. The GBM appeared to be diffusely thickened, with a mean value of GBM width determined by 16 direct measurements. The lamina rara interna spaces were segmentally widened, and a thin subendothelial basement membrane with occasional interposed mesangial cell processes was observed in a few capillary loops. The changes were interpreted as a consequence of altered intraglomerular hemodynamics. The endothelial cells were fenestrated (Figure 1A–C). Two conditions were considered in a differential diagnosis. One was oligomeganephronia of adult-onset type [6,7], which is a very rare entity and sometimes may be caused by *PAX2* mutations [6,8]. The other entity was obesity-related adaptive FSGS [1,9]. Because the genotyping procedure demonstrated a heterozygous missense mutation in exon 3 of the *PAX2* gene, the case was concluded as a *PAX2*-mutation-induced adult-onset oligomeganephronia and adaptive FSGS (FSGS7, OMIM: 616002), noting that severe obesity could contribute to the progression of kidney injury. The mutation did not shorten the length of the PAX2-protein (https://www.mutationtaster.org, last accessed on 15 October 2023).

#### 2.2.2. Investigations Performed after the Genotyping Procedure

The patient approved the screening for possible extrarenal manifestations of the *PAX2* mutation [10] through magnetic resonance urography, funduscopy of the eyes, a computed tomography scan of the temporal bones, and audiometry. All of them gave negative results. The oral glucose tolerance test excluded prediabetes. Seven months after the kidney biopsy evaluation, acute dyspnea emerged, and the echocardiographic investigation revealed dilative cardiomyopathy. The patient is now on complex antihypertensive and cardiovascular therapy.

The family history of the proband was thoroughly revised (Figure S1A). His mother was chronically hemodialyzed for chronic renal failure of unknown origin from the age of 45, and she passed away three years later (autopsy was not approved). The proband had a brother and two sons, and none of them had any symptoms of chronic renal disease. However, he had a daughter who died in the newborn period due to severe renal hypoplasia (2018; an autopsy was not agreed upon). In 2022, his wife was pregnant with identical male twins. An ultrasound evaluation of the pregnancy demonstrated oligohydramnios, and the pregnancy was terminated at the 21st week of gestation. The fetopathological examination revealed uniformly severe bilateral renal hypoplasia in both fetuses. The weight of the hypoplastic kidneys (0.2 g) was only one tenth of the aged and sized matched control. Investigations to explore the background of renal hypoplasia were not conducted at that time for unknown reasons.

**Figure 2:**
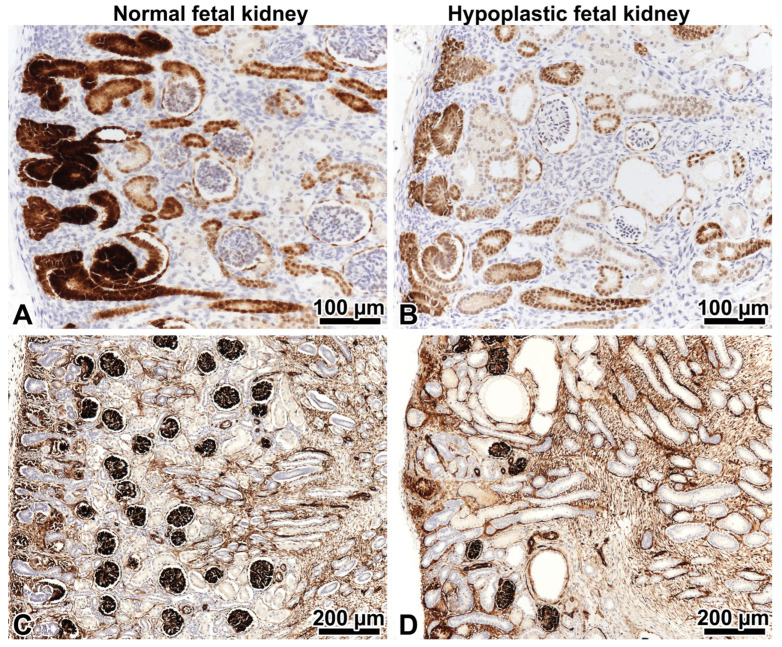
PAX2 and WT1 immunohistochemistry in fetal kidneys on strictly orthogonal cortico-medullary sections. Left: control kidney, right: hypoplastic kidney at 21 weeks gestation. (**A**,**B**) The expression of the PAX2 protein in the nephrogenic zone involves the cap mesenchyme, ureteral epithelium, and early nephron elements. The podocytes of mature glomeruli beneath the nephrogenic zone are negative. Note that the PAX2 expression is less intense in the hypoplastic kidney. This tells us it is abnormal. ×20. (**C**) Five to six generations of WT1-positive glomeruli beneath the nephrogenic zone in the control kidney. ×10. (**D**) Only 2–3 generations of WT1-positive glomeruli in the hypoplastic kidney are present. ×10.

The fetal kidneys were re-evaluated after the discovery of the *PAX2* mutation. The normal organization of the cortex, medulla, and renal pelvis was observed. Cortical tubular microcysts were present here and there. The nephrogenic zone comprised 18.1% of the cortical area, and 2–4 generations of mature, i.e., WT1-positive glomeruli were located under the nephrogenic zone (mean density: 18/mm^2^). In the control normal kidney, obtained from a pregnancy interrupted at week 21 of gestation because of a congenital heart defect, the nephrogenic zone comprised 24.6% of the cortical area, and 5–6 generations of WT1-positive glomeruli were located under the nephrogenic zone (mean density: 25/mm^2^). The PAX2 and WT1 expression of nephron segments beneath the nephrogenic zone were similar in the hypoplastic and control kidneys (Figure 2A–D). However, the expression of PAX2 in cap mesenchyme surrounding the ureteral bud tips, ureteral epithelium, and early nephron elements was less intense in the hypoplastic kidney, and the nephrogenic zone itself appeared to be thinner (Figure 2A,B).

#### 2.2.3. Comments

The PAX2-related disorder is characterized by autosomal dominant inheritance and, almost invariably, by renal hypoplasia, progressive renal failure in childhood, and optic nerve colobomas. The *PAX2* gene encodes a transcription factor expressed primarily during fetal development in the urogenital tract, eye, ear, and central nervous system. The gene organizes the descent of the mesonephric duct, the outgrowth of the ureteric bud from the mesonephric duct, and branching morphogenesis and arborization of the collecting system in cooperation with other transcription factors. Signals from each branch of the ureteric bud stimulate adjacent metanephric blastemal cells, and they differentiate into the elements of nephrons [11]. The homozygous inactivation of *PAX2* leads to bilateral renal agenesis, and this is incompatible with life. The heterozygous mutations affect the extent of branching morphogenesis, and in infants and children, they can manifest themselves in bilateral renal hypoplasia/hypodysplasia or multicystic dysplastic kidneys with progressive deterioration of renal function, and they may be associated with vesicoureteral reflux and/or other congenital abnormalities of the kidney and urinary tract [10]. Renal hypoplasia is characterized by small kidneys for a given age and hyperechogenicity on ultrasound examination, a reduced number of renal lobes seen macroscopically, and a markedly reduced number of nephrons showing compensatory hypertrophy and adaptive FSGS on histology. In some individuals, the proteinuria and deterioration of renal function appear only in adulthood, and ocular or hearing abnormalities may be absent. The evaluation of renal biopsy specimens in these patients commonly reveals adaptive FSGS [12] with or without the histologic diagnosis of oligomeganephronia.

In our proband, the consequences of *PAX2* mutation appeared to be restricted to the kidneys. A unique feature of the case was that we were able to follow the penetrance of the *PAX2* mutation in three generations of a family, manifesting itself with different degrees of severity, and we were able to examine the mutational consequences in the kidneys of identical twin fetuses at that gestational age when the *PAX2* gene is normally active. To the best of our knowledge, only one publication has so far correlated the histologic findings with the results of genetic testing in fetal kidneys with a *PAX2* gene mutation [13]. However, a detailed analysis of mutation-related changes in hypoplastic kidneys relative to appropriate controls was not the subject of that study. In the fetal kidney with *PAX2* mutation, a thinner nephrogenic zone, a decreased PAX2 protein expression in the nephrogenic zone, and a decreased number of glomeruli were found, demonstrating that the mutated PAX2 protein could not exert glomerulogenesis as intensely as the wild-type protein did in the control kidney. The hypoplastic fetal kidneys did not produce an appropriate amount of urine, and this led to oligohydramnios. The cause of renal hypoplasia in two generations of siblings remained obscure until a kidney biopsy evaluation of the proband revealed adult-onset oligomeganephronia and adaptive FSGS, so we conducted a mutational analysis of “FSGS” genes. The result of the genotyping procedure resolved the puzzle of renal disease affecting three generations within a family.

### 2.3. Patient 2

He was evaluated in 2019 owing to a significant degree of proteinuria, a preserved serum albumin level, slowly progressive chronic kidney disease stage III, and a family history of renal disease. The kidneys were slightly smaller than normal on ultrasound examination (longitudinal diameter of the right kidney: 90 mm; left kidney: 92 mm). Both kidneys’ parenchyma was slightly more echogenic than usual. He was born in the 38th week of gestation, and the birth weight could not be recalled. His father was chronically hemodialyzed for end-stage renal disease of unknown etiology from the age of 27 and received a kidney transplant twice. His father had two sisters, and they and their offspring were not affected by any renal disease. His father’s mother died of chronic renal failure of unknown origin. The proband lived in an infertile marriage (details of the cause not known). Two years after the renal biopsy evaluation, his wife got pregnant via in vitro fertilization with the proband’s sperm. The gestation ended with the successful delivery of a baby girl (Figure S1B).

#### 2.3.1. Renal Biopsy Findings and Results of a Genetic Analysis

Sclerosing glomerulopathy was observed, displaying an FSGS lesion in 11% of glomeruli. IF revealed focal-segmental mild IgM and intense C3 deposition in glomeruli. With EM, irregularities in GBM thickness were noted focally. However, basket-weave-like splitting and reduplication of lamina densa suggesting Alport nephropathy was not seen. Adult-onset genetic FSGS was assumed [14]. A mutational analysis identified a heterozygous missense mutation in the *ACTN4* gene and a hemizygous benign variation in the *CLCN5* gene. The daughter of the proband inherited the *ACTN4* mutation.

#### 2.3.2. Investigations Made after Performing the Genotyping Procedure

**Figure 3:**
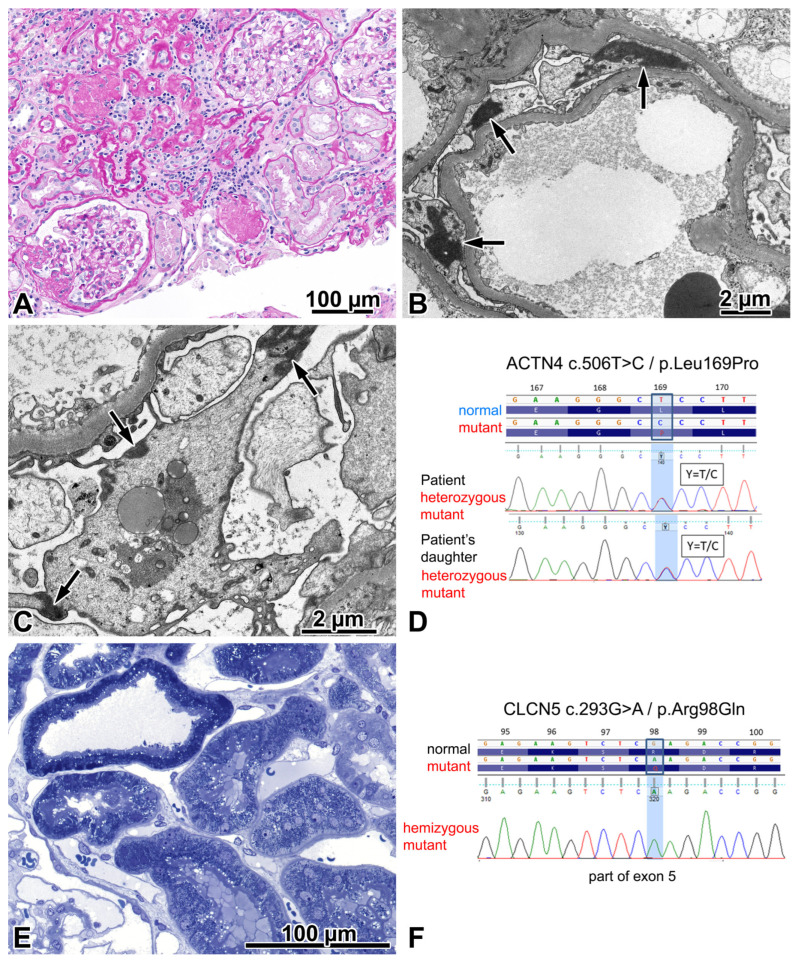
FSGS caused by an *ACTN4* mutation. (**A**) The photo depicts one segmentally sclerosed glomerulus, two remnants of globally sclerosed glomeruli, two patent glomeruli, and a significant degree of interstitial fibrosis and tubular atrophy. Periodic acid–Schiff, ×20. (**B**) Aggregates of highly electron-dense material (arrows) in the cytoplasm of podocytes, distinct for the mutation. ×3000. (**C**) Typically, these highly electron-dense aggregates are associated with the cell membrane (arrows), and they should be distinguished from electron-dense aggregates of lysosomal origin, located here in the centre of the podocyte. ×5000. (**D**) A heterozygous *ACTN4* mutation (exon 5; NM_004924.4:c.506T>C and NP_004915.2:p.Leu169Pro) was detected both in the proband and in his daughter. A single nucleotide change c.506T>C was identified in both. The Sanger sequence traces show the nucleotide change position (highlighted in blue). (**E**) The evenly distributed apical vesicles in proximal tubules do not lead us to suspect disturbed endocytosis in the apical endocytic apparatus. Semithin section, toluidine blue, ×40. (**F**) A hemizygous *CLCN5* mutation (exon 5; NM_001127898.2:c.293G>A and NP_001121370.1:p.Arg98Gln) was detected in the proband. A single nucleotide change c.293G>A was identified. The Sanger sequence traces show the nucleotide change position (highlighted in blue). The genetic, clinical, and morphologic features taken together indicated that this mutation had no renal relevance.

The podocytes and the apical endocytic–lysosomal apparatus of proximal tubules were re-examined electron microscopically to look for suggestive mutational abnormalities. Distinct electron-dense aggregates in podocyte cell bodies were found (Figure 3B,C). The small and large vesicles and dense tubules of apical endocytic apparatus in the proximal tubules looked normal (Figure 3E), and tubular calcium deposits were not observed histologically. Beta-2-microglobinuria was checked clinically and gave a negative result. The case was concluded as an *ACTN4*-mutation-induced familial FSGS (FSGS1, OMIM: 603278), and the *CLCN5* mutation was classified as benign. His kidney function slowly deteriorated, and 4 years after the renal biopsy evaluation, chronic hemodialysis treatment was initiated.

#### 2.3.3. Comments

Disease-causing *ACTN4* mutations commonly reside within the actin-binding domain of the encoded ACTN4 protein and increase the binding affinity of ACTN4 to filamentous actin, thus rendering the podocytes vulnerable to mechanical stress [15]. The mutation in our patient likewise affected the actin-binding domain. It was not detected in 360 Hungarian control samples, nor has it been included in the genome aggregation database (gnomAD), Exom Variant Server, ClinVar, Exome Aggregation Consortium, or HGMD databases. Nevertheless, the bioinformatical prediction of its effect (SHIFT, POLYPHEN, MUTATION TASTER) suggested a disease-causing mutation. Armed with the results of genetic testing, a PubMed literature search for renal biopsy abnormalities in ACTN4 mutants was performed. Afterwards, an electron microscopical study of five patients was found that described electron-dense aggregates in the cell bodies of podocytes, most likely composed of actin and mutant α-actinin-4, regarded as distinct for the mutation [16]. We repeated the study of podocytes with focus and came across these aggregates.

The *CLCN5* gene encodes a chloride and/or protein exchanger that is necessary for endosomal acidification and receptor-mediated endocytosis. Pathogenic mutations of the gene lead to Dent’s disease type 1 characterized by X-linked proximal tubulopathy, childhood onset, low molecular weight proteinuria, hypercalciuria, nephrocalcinosis and/or kidney stones, and a slow progression to end-stage renal disease in adulthood. Affected individuals may have an atypical phenotype, and FSGS may be the dominant feature in some patients [17]. Because the mutation found in our patient was classified as a variant of uncertain significance, we investigated the ultrastructural morphology of proximal tubules based on our experience of renal biopsy samples taken from three beta-2-microglobulinuric boys with a pathogenic *CLCN5* mutation. The Dent’s patients displayed distinct abnormalities in the apical endocytic–lysosomal apparatus, along with tubular calcium deposits and focal global glomerulosclerosis. However, the index patient showed normally appearing proximal tubules and no tubular calcium deposits, and a laboratory evaluation did not demonstrate any low-molecular-weight proteinuria, so the morphological and clinical findings supported the classification of the *CLCN5* variation as benign.

### 2.4. Patient 3

He was evaluated in 2019 owing to gradually declining renal function, proteinuria, microhematuria, hypertension, and hypercholesterolemia. The renal symptoms had been known for 15 years, and elevated blood pressure values had been known for 5 years. The question of a family history of hereditary glomerular disease remained unclear.

#### 2.4.1. Renal Biopsy Findings and a Post-Biopsy Evaluation for Alport Syndrome

**Figure 4:**
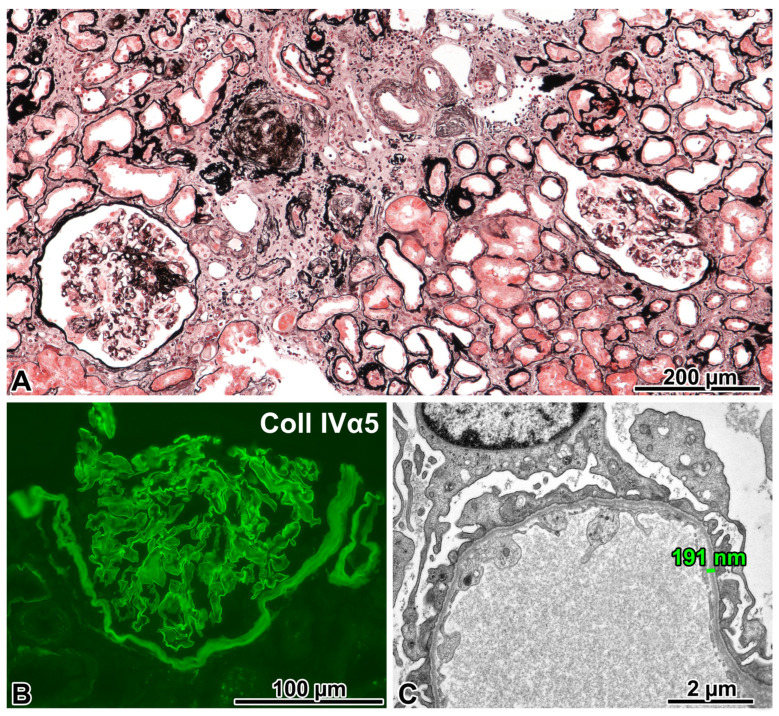

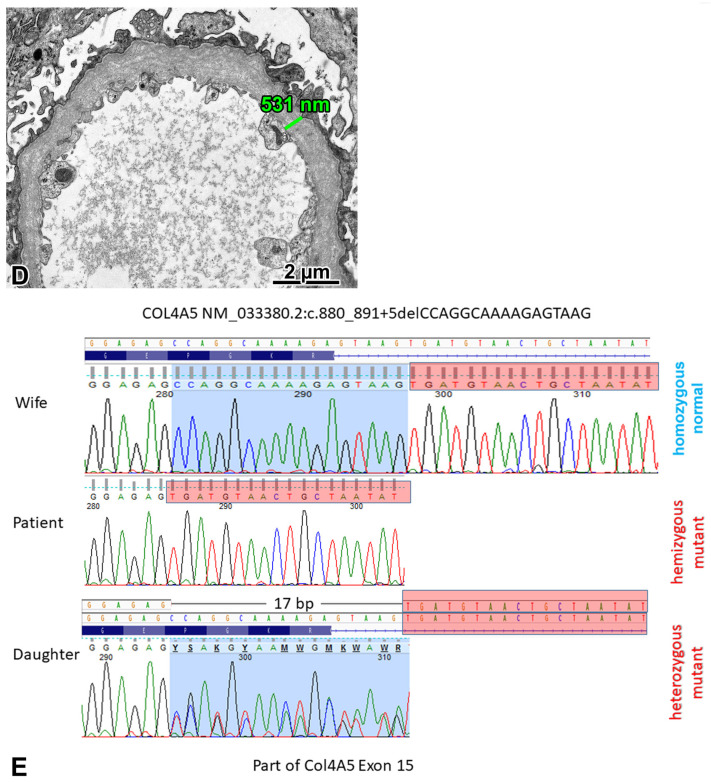
FSGS caused by a *COL4A5* mutation (X-linked Alport syndrome). (**A**) Two glomeruli with segmental sclerosis of different degrees, remnants of two globally sclerosed glomeruli, and marked interstitial fibrosis and tubular atrophy can be seen in this visual field. Jones, ×10. (**B**) The anti-α5 chain(IV) antibody cocktail stained the glomerular basement membrane (GBM), the basement membrane of Bowman’s capsule, and some tubules. Immunofluorescence, cryostat section. ×40. (**C**) The GBM was uniformly thin in two glomeruli. ×6000. (**D**) The diffuse lamination and splitting of the GBM (“basket-weave” pattern) was noted in one glomerulus. ×5000. (**E**) A hemizygous *COL4A5* mutation (exon 15; NM_033380.2:c.880_891+5delCCAGGCAAAAGAGTAAG) was detected in the proband. His daughter is a heterozygous carrier for this 17-nucleotide-long deletion, but his wife did not have it. The Sanger sequence traces show the nucleotide change position (highlighted in blue).

Pronounced chronic sclerosing changes, and an FSGS not otherwise specified variant in patent glomeruli, mild glomerulomegaly, and interstitial foamy cells were observed light microscopically (Figure 4A). The IF evaluation was negative for immune complexes. The antibody cocktail against the α5 chain stained the GBM (Figure 4B), the basement membrane of Bowman’s capsule, and some of the tubules. Three glomeruli were examined electron microscopically. Two exhibited diffusely thinned GBM (Figure 4C). In the third one, the basket-weave appearance of GBM was noted (Figure 4D). The FPE was focal. Because the EM findings strongly suggested Alport syndrome, the patient was investigated for extrarenal manifestations. Bilateral hypoacusis was diagnosed shortly after the renal biopsy. No Alport-related ocular changes were found. Regarding family involvement, the urinalysis performed on the patient’s daughter revealed microhematuria (Figure S1C). The patient is now close to end-stage renal disease 4.5 years after the kidney biopsy evaluation.

#### 2.4.2. Genetic Analysis

The proband carried a hemizygous mutation, a 17-nucleotide-long deletion in the *COL4A5* gene. His daughter proved to be a heterozygous carrier for the deletion, but his wife did not carry it. Considering the hearing loss of the proband, X-linked Alport syndrome (XLAS) was diagnosed.

#### 2.4.3. Comments

A case of XLAS with an unusual immunophenotype and age of onset was presented. Regarding the clinical aspects, although microhematuria and proteinuria had already been documented by the age of 28, the patient did not develop proven chronic kidney disease for another thirteen years. Because the EM findings in one glomerulus were consistent with Alport nephropathy and the other two glomeruli displayed diffusely thinned GBM, we conducted a search for the *COL4A5* mutation, with a positive result. To date, more than 1500 mutations affecting the *COL4A5* gene are known to cause XLAS, with a wide range of effects. Each collagen IV chain contains three domains: a short 7S domain at the N-terminal region, a long, collagen-like domain in the middle, and a non-collagenous (NC) domain at the C-terminal. Frame-shift mutations, as well as other abnormalities affecting the NC domain, typically result in truncated gene products, leading to classic early onset with a severe clinical course. However, missense mutations and in-frame deletions generate minor changes in the molecule, corresponding to a mild phenotype [18].

As for our patient, the genetic testing identified a previously unreported deletion of the *COL4A5* gene in exon 15, which was also present in his daughter. Because this anomaly, to the best of our knowledge, cannot be found in any registers, including the gnomAD, its effect can only be predicted by means of bioinformatics, e.g., using VarSome. The mutation causing a splice junction loss was predicted to be most likely pathogenic following the guidelines of the American College of Medical Genetics and Genomics. Based on the MutationTaster prediction, at the translation level, four amino acids (a Pro-Gly-Lys-Arg sequence at position 294–297) have probably been deleted from the mutant protein, which is otherwise identical to the normal product of the *COL4A5* gene. As for the IF findings, it is known that XLAS cases may display preserved positivity for collagen IV α5 chains [19]. According to the manufacturer, the anti-α5 antibody cocktail is specific to the imperfection III (clone H53) and NC1 domain (clone B51) of the α5 chain. Imperfection III is an epitope defined by a sequence of five amino acids. This region is located at position 251–255 in exon 13, which is not far away from the mutated area. These exons encode the central, collagen-like domain of the protein, which is characterized by numerous Gly-X-Y repeats that are responsible for a helix formation. We think that the deleted area may induce a disruption in protein folding and mask the epitope of clone H53. The target epitope of clone B51 is in the NC1 domain, and it is composed of approximately 230 amino acids at the C-terminal end. Based on the mild clinical course, we assume that this protein region was not affected by the mutation, which is a plausible explanation for the maintained positivity for the α5 chain on IF. Next, we should consider the limitations of our observations. As the demonstrated deletion encompasses not only exon 15, but also five nucleotides from the intron following it, we cannot rule out the possibility of alternative splicing. This could be investigated by means of a transcriptional analysis. Regrettably, we could not analyze the RNA expression profile in this patient.

In conclusion, we identified an XLAS patient with a new, to-date-unreported, likely pathogenic mutation of the *COL4A5* gene. Although most XLAS patients with a typical clinical presentation are diagnosed nowadays with a genetic examination without performing a renal biopsy procedure, atypical, late-onset cases are biopsied, and the ultrastructural abnormalities of GBM suggest the need for genetic testing for the *COL4A5* mutation. A positive result will establish the diagnosis.

## 3. Discussion

### Lessons Learned from the Case Presentations

Adult-onset genetic FSGS is a rare disease [20], and the time required to obtain a genetic diagnosis from the initial manifestation of the kidney disease might exceed a decade [21]. In our patients, it took 6 years, 8 years, and 15 years, respectively, for it to be conclusive. The appropriate clinicopathologic classification of FSGS types facilitates the proper patient selection for genetic testing and cuts down the time for arriving at a genetic diagnosis. In the publication from the Mayo Clinic [2], FSGS in non-diabetic adults was classified into primary, secondary with known cause, secondary without known cause, and undetermined. The detection rate of monogenic FSGS was noticeably high in individuals with undetermined FSGS and individuals with secondary FSGS without an identifiable cause. The family history of kidney disease and absence of nephrotic syndrome were strong predictors for finding a causative genetic variant. Table 1 lists certain clinical and pathologic features that increase the likelihood of adult-onset FSGS. If any of these are present, genetic testing should be conducted [3,16,21,22]. The testing usually identifies either mutations in genes encoding podocyte proteins, such as NPHS2, ACTN4, or INF2, or mutations in genes encoding GBM proteins, such as COL4A5.

In retrospect, the phenotypic features of the presented cases were characteristic of the mutation identified. In the severely obese 29-year-old patient, the paucity of enlarged glomeruli, and the positive family history affecting three generations, favored a PAX2-related disorder. In the 31-year-old patient, the onset of renal symptoms in early adulthood, the non-nephrotic proteinuria, the positive family history, and the distinct electron-dense aggregates in podocytes were consistent with an *ACTN4* mutation. In the 43-year-old patient, the male gender, the proteinuria associated with microhematuria, the bilateral hearing loss, and the splitting and lamination of GBM all mean it cannot really be any disease other than XLAS.

The results of genotyping resolved the puzzles in the phenotypic manifestations. The thorough revision of the family history for kidney disease in Patient 1 revealed congenital renal hypoplasia of unknown origin that affected two generations of siblings of the proband. The mutation identified and the subsequent immunohistochemical analysis of the PAX2 protein expression in fetal kidneys explained why these were hypoplastic. After finding the *ACTN4* mutation, cytoplasmic electron-dense aggregates in podocytes distinct for the entity were sought, and by doing so, we should recognize the aggregates in a future case. The molecular features of the *COL4A5* mutation accounted for the discrepancy between the characteristic ultrastructural GBM alterations of Alport nephropathy and the immunohistochemical positivity of the α5 chain staining on IF. Lastly, we should stress the importance of positive genotyping results in clinical nephrology practice for the prognosis of the disease, treatment decisions, and family counseling.

## 4. Materials and Methods

### 4.1. Evaluation of Renal Biopsy Samples

These were evaluated through standard light microscopical stainings of formalin-fixed, paraffin-embedded tissue samples, direct immunofluorescence (IF) on cryostat sections (FITC-conjugated antibodies to IgG, IgA, IgM, C3, C1q, kappa, lambda, fibrinogen), and electron microscopy (EM, fixation in 3% phosphate-buffered glutaraldehyde supplemented with dextran, post-fixation in 1% osmium tetroxide, and embedding to Epon resin). The glomerular density was calculated by dividing the number of patent and globally sclerotic glomeruli by the total cortical area. Glomerulomegaly was diagnosed if the mean diameter of glomeruli exceeded 220 µm, measured in at least 5 patent glomeruli with the vascular pole in the section plane. Glomerulosclerosis, tubular atrophy, interstitial fibrosis, and arteriosclerosis were graded semiquantitatively, as recommended [23]. FPE was classed as diffuse if more than 80% of glomerular capillary loops were involved [2].

### 4.2. Additional Immunostainings and Measurements

The histopathological evaluation of formalin-fixed, paraffin-embedded fetal kidneys taken from the offspring of Patient 1 was supplemented with PAX2 (1:100, clone EP235, Bio SB, Santa Barbara, CA, USA) and WT1 (1:300; clone 6F-H2, Cell Marque, Sigma-Aldrich Company, Merck KGaA, Darmstadt, Germany) immunostainings. The BOND Polymer Refine Detection system (Leica Biosystems, Deer Park, IL, USA) was used for the visualization. The 3DHISTECH PANNORAMIC VIEWER software package (Version 1.15.4., 3DHISTECH Kft., Budapest, Hungary) and digitalized slides were used to calculate the density of mature glomeruli (number/mm^2^; podocytes positive with WT1, and negative with PAX2), and to assess the area of the nephrogenic zone relative to the renal cortex (the nephrogenic zone plus the zone of WT1-positive glomeruli, measured in mm^2^).

The expression of the α2 chain and the α5 chain of type IV collagen on frozen sections from Patient 3 was investigated with fluorochrome-conjugated monoclonal antibodies to the α2 chain and the α5 chain (catalogue number: CFT-45325, Shigei Medical Research Institute, Yamada, Japan). According to the manufacturer, the anti-α5(IV) antibody cocktail is specific to imperfection III of the α5(IV) chain (clone H53; rat IgG2a/kappa) and the NC1 domain of the α5(IV) chain (clone B51; rat IgG2a). The clone H25 (rat IgG1a/κ) is specific to imperfection XIII of the α2(IV).

### 4.3. Genetic Analyses

Clinical exome sequencing was performed in the patients. Genomic DNA was prepared from blood samples using the MagCore Genomic Whole Blood Kit (RBC Bioscience, New Taipei City, Taiwan). Genomic capture was carried out with the TrusightOne Expanded CES Kit (Illumina, San Diego, CA, USA). The following 45 genes were tested: *ACTN4* (19q13.2), *ALG1* (16p13.3), *ANLN* (7p14.2), *ARHGAP24* (4q21.2-q21.3), *ARHGDIA* (17q25.3), *CD2AP* (6p12.3), *CLCN5* (Xp11.23), *COL4A3* (2q36.3), *COL4A4* (2q36.3), *COL4A5* (Xq22.3), *COL4A6* (Xq22.3), *COQ2* (4q21.22-q21.23), *COQ6* (14q24.3), *COQ8B* (19q13.2), *CRB2* (9q33.3), *CUBN* (10p13), *DGKE* (17q22), *ITGA3* (17q21.33), *ITGB4* (17q25.1), *ITSN1* (21q22.11), *ITSN2* (2p23.3), *KANK1* (9p24.3), *KANK2* (19p13.2), *KANK4* (1p31.3), *LAMA5* (20q13.33), *LAMB2* (3p21.31), *LMX1B* (9q33.3), *MAGI2* (7q21.11 ), *MYH9* (22q12.3), *MYO1E* (15q22.2), *NPHS1* (19q13.12), *NPHS2* (1q25.2), *NUP107* (12q15), *NXF5* (Xq22.1), *PAX2* (10q24.31), *PDSS2* (6q21), *PLCE1* (10q23.33), *PTPRO* (12p12.3), *SCARB2* (4q21.1), *SMARCAL1* (2q35), *TP53RK* (20q13.12), *TRPC6* (11q22.1), *WDR73* (15q25.2), *WT1* (11p13), and XPO5 (6p21.1). Massively parallel sequencing was carried out using the Miseq Sequencer (Illumina, San Diego, CA, USA) in combination with the MiSeq™ 300 v2 Output Kit (2 × 150 bp). Raw sequence data analyses, including base calling, de-multiplexing, alignment to the hg19 human reference genome (Genome Reference Consortium GRCh37), and variant calling, were performed using an in-house bioinformatics pipeline. For variant filtration, all of the disease-causing variants reported in HGMD and ClinVar, along with all of the variants with a minor allele frequency of less than 1% in the ExAc Database, were considered. Variants that possibly impair the protein sequence, like the disruption of conserved splice sites, missense, nonsense, read-throughs, and small insertions/deletions, were prioritized. All of the relevant inheritance patterns were considered, and the candidate pathogenic mutations were verified through PCR amplification and Sanger sequencing for the individuals studied.

## Figures and Tables

**Table 1:** Clinicopathological features suggestive of adult-onset genetic FSGS.

Initial manifestation started before age 25
Absence of the nephrotic syndrome
Family history of kidney disease
Extrarenal manifestations (hearing loss, eye abnormalities, etc.)
Undetermined FSGS or secondary FSGS without an identifiable cause
Oligomeganephronia on LM or abnormalities in podocytes * or GBM ** on EM

Abbreviations: EM: electron microscopy, FSGS: focal segmental glomerulosclerosis, GBM: glomerular basement membrane, LM: light microscopy. * *ACTN4* mutation: cytoplasmic electron-dense aggregates attaching to the cell membrane, *INF2* mutation: irregular and jagged foot processes focally, often with longitudinal actin bundles. ** Irregularity, a basket-weave appearance, thinning, scalloping, wrinkling, microparticles.

## Data Availability

Sequence data are unavailable due to privacy and ethical restrictions.

## Supplementary Materials

The supporting information can be downloaded at: https://www.mdpi.com/article/10.3390/ijms242417489/s1.
