# Supplementary material for: Phenotype–Genotype Correlations in Three Different Cases of Adult-Onset Genetic Focal Segmental Glomerulosclerosis
Source: Int J Mol Sci. 2023 Dec 14;24(24):17489. doi: 10.3390/ijms242417489 (PMC10743622; doi:10.3390/ijms242417489)
Supplement: Supplementary file 1 [file ijms-24-17489-s001.zip › ijms-2760309-supplementary.pdf]

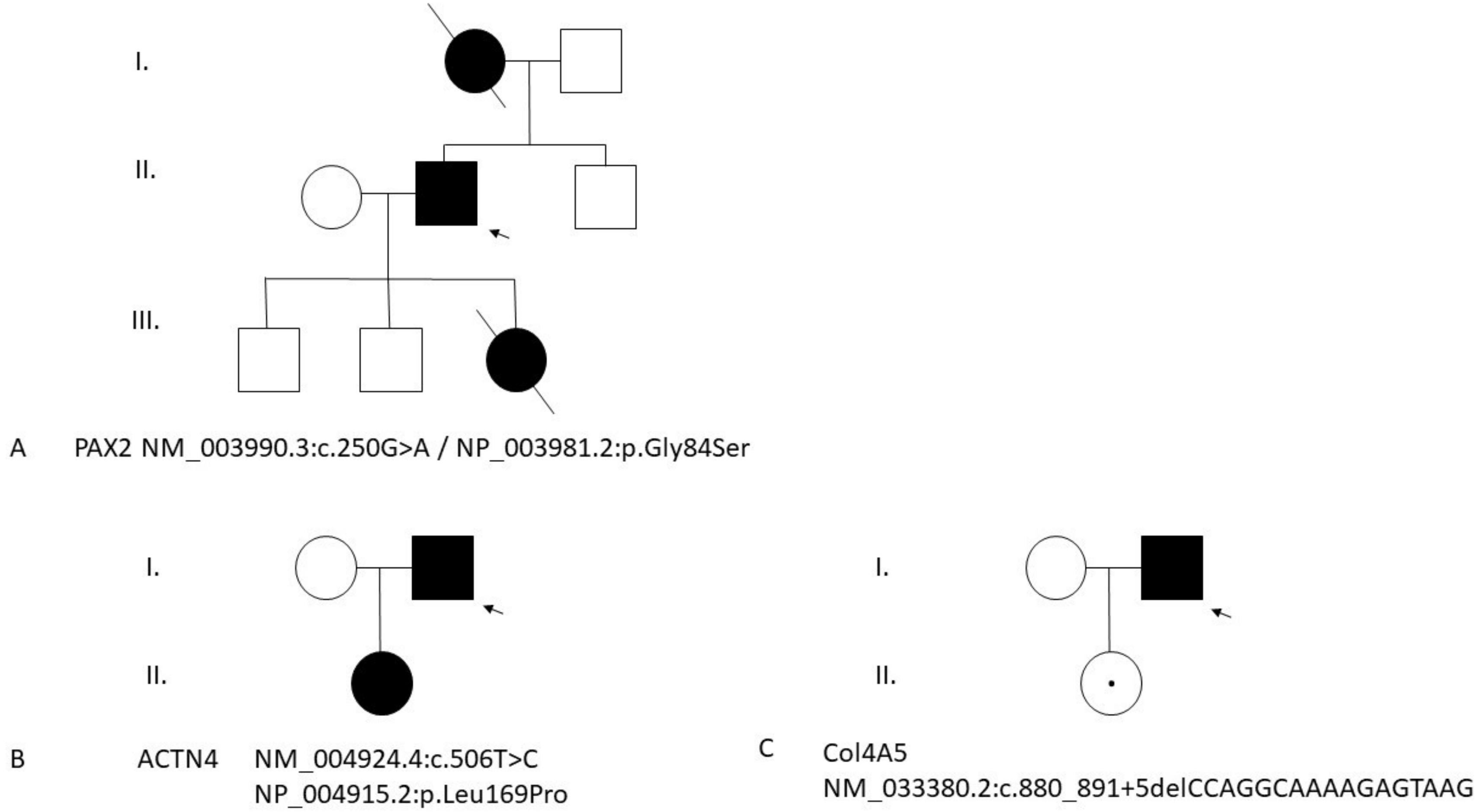

Figure S1. Genealogical trees of the investigated FSGS families.
A, Genealogical tree of Family of Patient 1. **B**, Genealogical tree of Family of Patient 2. **C**, Genealogical tree of Family of Patient 3.
Generations are denoted by Roman numerals. Unfilled symbols indicate unaffected, and filled symbols indicate affected family members. Circles represent females, squares represent males. Deceased individuals are indicated by crossed symbols and arrows mark Probands.

Table S1. Adult-onset genetic FSGS. Medical history and clinical data at the time of the kidney biopsy

**Race, gender, age (year)**	**Mutated gene**	**Proteinuria (g/d)**	**Micro-hematuria**	**eGFR (mL/min/1.73 m²), se-creat (mol/L)**	**Renal ultrasound**	**BMI (kg/m²)**	**Extra-renal involvement**	**Kidney disease history, time to diagnosis (year)**	**Follow up**	**Family history**
Roma male, 29	*PAX2,* exon 3, paired box domain	4, NS not present	No	28, 276	Smaller-sized kidneys, normal thickness, hyperechogenic	44, obese from childhood	Negative for optic nerve coloboma and bilateral hearing loss; normal renal pelvis, ureter, and bladder *	Proteinuria known for 6 years, hypertension for a couple of years TD: 6 years	OGTT: negative, DCM 7 months after the Bx, eGFR 12 months after the Bx: 28 ml/min	Mother: ESRD of unknown etiology Deceased neonate daughter and male twin fetuses **: bilateral renal hypoplasia
Caucasian male, 31	*ACTN4*, exon 5, CH2 domain of actin-binding domain *(CLCN5,* exon 5)	3 to 5.5, NS not present β-2-MG: neg.	No	39, 190	Smaller-sized kidneys, normal thickness, hyperechogenic	24.4	No	CKD, proteinuria, hypertension known for 7 years TD: 8 years	ESRD within 4 years	Father: ESRD of unknown etiology Daughter: heterozygous mutant, followed clinically
Caucasian male, 43	*COL4A5,* exon 15	2.73, NS not present	Yes	33, 205	Smaller-sized kidneys, normal thickness, hyperechogenic	26.6	Bilateral hypacusis; hearing aid from age 43; no Alport-related ocular changes	Age 28: microhematuria, proteinuria Age 38: hypertension Age 41: CKD TD: 15 years	53 months after the Bx: close to ESRD	Father: not known to have chronic renal disease Mother: chronic renal disease, not specified Heterozygous mutant daughter: microhematuria

Viral- and drug-associated secondary FSGS were excluded by using clinical and serologic evaluations.

Abbreviations: β-2-MG: beta-2-microglobulin, BMI: body mass index, Bx: biopsy, CKD: chronic kidney disease, DCM: dilative cardiomyopathy, eGFR: the estimated glomerular filtration rate, ESRD: end-stage renal disease, g/d: gram/day, NS: nephrotic syndrome, OGTT: oral glucose tolerance test, se-creat: serum creatinine, TD: the overall time to a genetic diagnosis from the first manifestation of a renal disease in years. * Examined via intravenous urography, and abdominal ultrasound. ** Terminated pregnancy at the 21st week of gestation because of oligohydramnios.

Table S2. Adult-onset genetic FSGS. Kidney biopsy findings

**Gene**	**GGS (%)**	**FSGS (%)**	**Glom. Density (../mm²)**	**Glom. Diameter (µm)**	**IFTA (%)**	**Interst. foamy cells**	**TRCS**	**Podocyte FPE (%)**	**Podocyte cell body abnormality**	**GBM**	**Diagnosis**
PAX2	20	44, NOS variant	1.5*	308, range 284 -328	55	Yes	9	50	Microvillous transform-ation	Diffusely thickened, mean 533 nm, range 514-766; new subendo-thelial BM focally	OMN and adaptive FSGS due to the *PAX2*-mutation in the severely obese patient
ACTN4 (*CLCN5*)	43	11, Tip domain location	2.8	176	40	No	7	40	Electron-dense aggregates	Focally thin (264-286 nm), focally thick (412-430 nm)	FSGS due to *ACTN4*-mutation<br><br>No evidence of a C*LCN5* mutation *
COL4A5	23	41, NOS variant	2.5	218	45	Yes	7	40	Microvillous transform-ation	Diffusely thin in 2 gl-i, diffusely split and laminated in 1 gl<br>IF: reacted with an anti-alpha-5 moAB cocktail and anti-alpha-2 moAB	X-linked Alport nephropathy

BM: basement membrane, FPE: foot process effacement, FSGS: focal-segmental glomerulosclerosis, GBM: glomerular basement membrane, GGS: Global glomerulosclerosis, gl: glomerulus, Glom.: glomerular, IF: immunofluorescence, IFTA: interstitial fibrosis and tubular atrophy, Interst.: interstitial, moAb: monoclonal antibody, NOS: not otherwise specified, OMN: oligomeganephronia, TRCS: total renal chronicity score: graded chronic lesions in renal tissue compartments, with a maximum score of 10.

* Reference value: 4.07/mm², range 3.21–4.92; assessed in thin basement membrane nephropathy cases [7].
